# Smartphone addiction prevalence, patterns of use, and experienced musculoskeletal discomfort during the COVID-19 pandemic in a general Iranian population

**DOI:** 10.1186/s12889-024-17654-3

**Published:** 2024-01-11

**Authors:** Hamid Reza Mokhtarinia, Maryam Heydari Torkamani, Nasim Farmani, Charles Philip Gabel

**Affiliations:** 1 Department of Ergonomics, University of Social Welfare and Rehabilitation Sciences, Tehran, Iran; 2 Iran Welfare Organization, PhD of Social Work, Tehran, Iran; 3 Access Physiotherapy, Coolum Beach, Sunshine Coast, QLD Australia

**Keywords:** Smartphone, Social networking, Pain, Prevalence, Risk factors

## Abstract

**Background:**

Smartphone usage is an essential everyday tool in Iran, however problematic use has escalated and become a concern for the Iranian health policy system, particularly during and following the COVID-19 Pandemic. This study’s aim was investigation of the prevalence of smartphone addiction, patterns of use, and the relationship to specific demographic characteristics and associated musculoskeletal disorders during the COVID-19 pandemic.

**Methods:**

A descriptive-analytical correlational study recruited participants from a population of convenience (*n* = 2344) who were smartphone owners with > 1 year of use. For demographic information an electronic self-report questionnaire collected age, sex, marital status, usage for daily hours, and patterns. To assess addiction levels, the ‘Smartphone Addiction Scale-short version’ (SAS-SV) patient-reported outcome measure was used (cut-off = 31). For experienced discomfort, the Extended Nordic Musculoskeletal Questionnaire (ENMQ) was used.

**Results:**

The participants (female = 66.6%, *n* = 1561, mean age = 29.07 ± 12.34 years, range 6–60 years) smartphone use averaged 5.75 ± 3.44 h/day. The general prevalence of smartphone addiction was 46.16% (females = 46.06%, males = 46.36%; married = 44.5%, single = 47.63%). School students had the greatest addiction (53.2%) and those with a higher education to or above a Master’s degree were the lowest (39.38%). The highest pattern of use was for social networks at 89.1% of participants (female = 88.34%, male = 90.54%). The areas of highest reported discomfort were the eyes (43.5%) and neck (43.3%). A significant correlation was found between smartphone addiction and hours of daily usage, and the amount of usage increased during the COVID-19 pandemic period.

**Conclusion:**

A high level of smartphone addiction in the Iranian population was found to have occurred during the COVID-19 pandemic. Those most affected were unmarried individuals and school students, with the predominant areas being the eyes and neck. Health decision-makers should consider these findings when developing recommendations and plans for public health, particularly those focused on students.

## Background

The coronavirus disease 2019 (COVID-19) was declared a global pandemic by the World Health Organization (WHO) on March 11, 2020 [1]. The consequence for individuals and societies was that many countries within the global community implemented serious restrictions to prevent further spread of the disease. Due to the COVID-19 outbreak, people’s lives transformed significantly [2] and many organizations shifted the permitted and recommended working practices of millions of people to the home setting and/or the use of remote working circumstances to limit contact between individuals [3]. Iran was no exception, with all age groups and occupations affected, with the remote work model implemented that required the internet and other forms of technologically based communication. Different domains, including healthcare, education, and public services, all had their daily life notably dependent on technology to enable and facilitate their work, social and personal demands [4, 5]. Consequently, technology use during the COVID-19 pandemic in the Iranian workers’ daily lives occurred in ways that had not been previously envisaged or planned, while their ergonomic and individual personal health requirements similarly received minimal planning [4].

Smartphone technology, as a developed class of the cellular telephone, has been widely accepted throughout the globe due to its combined properties of mobile telephone and computing function combined into a single unit and equipment item. The potential of smartphone usage in different domains of communication, information processing, and entertainment, facilitated it to be the most practical device that met the majority of the individual’s needs within a remote work, study, and home setting during the COVID-19 pandemic [6]. This included online study courses from home being initiated for school children, students, and adolescents, instead of conventional school models [7]. The imposition of lockdowns subsequently led to an increase in the time spent using digital technology devices that included smartphones, screens, tablets and laptops [8, 9]. Healthcare providers also adopted these technologies and computing alternatives as a safer means of providing medical care during the COVID-19 pandemic period [10]. Teachers used the devices for arranging discussion sessions and assessing student performance [11]. Many office-based workers were advised and required to work from home and subsequently employed digital technology, especially smartphones [12].

These behavioral changes related to smartphone use were already well recognized as being associated with reduced physical activity, sedentary behavior, and sleep disturbance [13]. Additionally, there was an increased presence of musculoskeletal disorders and a range of other adverse physiological and psychological effects [14]. The presence of COVID-19, along with the subsequent pandemic and introduction of altered social contact and lockdowns, led to an increased tendency towards the overuse of internet-based technology and digital devices, with the smartphone often becoming the first-choice digital device for many individuals. Consequently, the public health measures and restrictions placed on the global population during the COVID-19 pandemic resulted in an immediate uptake and widespread use of smartphones, with the consequence of a concurrent ‘smartphone pandemic’ [15]. Smartphones were already well recognized as being most commonly used for browsing the internet, digital game-play, entertainment, and social networking [16]. Research studies prior to the COVID-19 pandemic demonstrated that smartphone use patterns were directly associated with smartphone addiction [17, 18] with the reported addiction at 23% in the general population [19], 24.9% in school students [17] and 13.5% in adolescents [18].

During the COVID-19 pandemic, the pattern of usage was definitively altered with circumstances for the potential of such addictive behavior increased due to the additional available time, limited social interactions, and lack of the pre-pandemic routine of daily activity [20, 21]. This increase during the pandemic was reported in the general population at a prevalence of 36.7% [22]. This was also representative of university students at 37.4% [23] but notably lower than the 53.3% recorded for children [24], 52.8% for medical students [25], and 46.7% in Italian children and adolescents [26].

To our knowledge, no prior comprehensive studies have examined the impact of COVID-19 confinement on smartphone usage patterns, related musculoskeletal disorders (MSDs), and physical activity levels in a general mixed population of varied age, sex, marital status, and education levels. Consequently, this current study was conducted to investigate the prevalence of MSDs and the level of physical activity while using a smartphone during the COVID-19 pandemic. Further, the pattern of smartphone use among these different groups was compared and the relationship between smartphone addiction and the prevalence of MSDs was investigated.

## Methods

### Participants and procedure

This online cross-sectional survey was conducted between May 2021 and February 2022, from a sample of convenience taken from the Iranian general population that included a total of *n* = 2344 (females = 1561, males = 783, age 6–60 years). Inclusion criteria were that participants had their own smartphone and a history of smartphone use of > 1 year.

The informed consent and the related research questionnaires were developed in an electronic format using the Porsline online survey system (https://survey.porsline.ir). The related electronic link was shared through social media platforms (WhatsApp, Telegram) and email. All participants were required to complete the informed consent and three self-reported rating questionnaires. Progression to the online questionnaires was digitally restricted until informed consent was completed. For children under the age of 16, written informed consent was obtained from their parent or guardian prior to the completion of the survey questionnaires. Institutional Review Board approval was obtained from the Ethics Committee of the University of Social Welfare and Rehabilitation Sciences (USWR), Iran, Tehran (IR.USWR.REC.1400.197).

### Survey instruments and questionnaires

In this cross-sectional study, three questionnaires were used to gather related data on the individual participants’ sociodemographics, musculoskeletal disorders, and level of smartphone addiction.

#### Sociodemographic questionnaire

A self-reported questionnaire was provided to the participants for their age, sex, level of education, daily smartphone usage (hours per day), and smartphone usage pattern [25, 26].

#### Smartphone addiction scale-short version (SAS-SV)

The SAS-SV, developed by Kwon et al. [27], was validated in an Iranian population in 2020 by Fallahtafti et al. [28] with the official translated and culturally adapted Persian version in 2023 by Mokhtarinia et al. [29]. This Persian SAS-SV version was used to measure the level of participant smartphone addiction. The SAS-SV is a 10-item patient-reported outcome measure (PROM) that is constructed based on a 6-point Likert scale (1 = strongly disagree, 2 = disagree, 3 = weakly disagree, 4 = weakly agree, 5 = agree, 6 = strongly agree) with a total score range of 10–60 [27]. According to the original SAS-SV recommendations, the threshold or cut-off score to detect pathological smartphone usage is for males ≥ 31 and females ≥ 33 [27, 30].

#### Extended nordic musculoskeletal questionnaire (ENMQ)

The ENMQ was developed by Dawson et al. [31] and is widely used in occupational populations to evaluate the prevalence, onset, and outcomes of MSK pain and conditions in nine body regions (the neck, shoulder, upper back, elbow, wrist/hand, low back, hip/thigh, knee, ankle/foot) over the preceding 12 month period, the last four weeks, and on the day of administration [31]. In this study, the upper limb (shoulder, upper back, elbow, wrist/hand) and the spine (neck, upper back) components of the ENMQ were utilized to detect the presence of MSD conditions during the periods of the individuals’ smartphone usage. We also added an extra dichotomous question at the end of the ENMQ pertaining to perceived eye discomfort with the simple yes/no response option. This additional question was placed in the given position to minimize participant burden rather than adding an extra single-item questionnaire. This follows the precedent of previous studies [11, 32, 33] that also used a single dichotomous eye-strain question that reflected the ENMQ response options.

### Statistical analysis

Descriptive statistics including mean and standard deviation (SD) of all continuous variables for general participants and based on the demographic characteristics were computed. Further, frequency and percentage were computed for categorical variables. To evaluate the mean differences in SAS-SV score between different categories including age, gender, and educaton, an independent t-test or one-way ANOVA and related post-hoc Bonferroni comparisons were conducted. The correlation between the smartphone addiction level and MSDs in different body regions in general, and according to age, gender and education, were evaluated by Pearson’s chi-squared test. Multivariable logistic regression analyses (forward LR) were used to assess related factors for smartphone addiction during the pandemic period. All statistical data analyses were performed using SPSS Version 16.0 for Windows. A significance level was set at 0.05 for all data.

## Results

### Demographic characteristics

The socio-demographic characteristics are presented in Table 1. A total of 2344 participants (mean age = 29.07 ± 12.34 years) were recruited for this study. Females were more highly represented at 66.6% (*n* = 1561) of which 40.4% were married (*n* = 946), and 65.9% (*n* = 1082) were < 35 years old; 46.16% (*n* = 1082) of participants were above the SAS-SV addiction cut-off score, 24.4% (*n* = 571) were school students, 24.9% (*n* = 583) were undergradautes, and 50.8% (*n* = 1190) were post-graduates. In general, most participants (57.2%) reported their smartphone usage at ≥ 5 h/day (average = 5.75 ± 3.44). In the addicted and non-addicted groups, the mean duration of smartphone use in hours/day was respectively 7.05 ± 3.53 and 4.63 ± 2.92.

**Table 1:** Demographic profile and general characteristics of participants (*n* = 2344)

Variable	Category	Number (percentage)
Gender	Female	1561 (66.6)
	Male	783 (33.4)
Addiction level	Addicted	1082 (46.16%)
	Non-addicted	1262 (53.84%)
Education level	School student	571 (24.4)
	Diploma- Undergraduted student	583 (24.9)
	Bachelor’s Degree	490 (20.9)
	Msc degrre and above	700 (29.9)
Age (year)	0–14	272 (11.6)
	15–24	727 (31)
	25–34	546 (23.3)
	35–44	500 (21.3)
	≥ 45	299 (12.8)
Martial status	Single Marriage	1398 (59.6) 946 (40.4)
Daily duration of use (hour/day)	0–4 h/day ≥ 5 h/day	1004 (42.8) 1340 (57.2)

### Smartphone addiction prevalence, musculoskeletal pain, and pattern of use

The descriptive results demonstrated the overall prevalence of smartphone addiction at 46.16%, according to the SAS-SV cut-off score. The prevalence of smartphone addiction in male, female, married, and single participants were respectively 46.36%, 46.06%, 44.50%, and 47.63%. The SAS-SV mean, standard deviation (SD), and prevalence of addiction are presented in Table 2.

**Table 2:** Prevalence of smartphone addiction and SAS-SV score difference classified by age, gender, and education

Variables		Mean (SD) of SAS-SV score	Smartphone addiction Prevalence n (%)	SAS-SV score diffrence, F (*P*-value)
Total (*n* = 2344)		31.99 ± 11.19	1082 (46.16)	-
Age (year)	0–14	32.75 ± 12.36	135 (49.63)	1.77 (0.13)
	15–24	32.65 ± 11.70	351 (48.28)	
	25–34	31.20 ± 10.56	232 (42.49)	
	35–44	31.62 ± 10.52	227 (45.4)	
	≥ 45	3177 ± 10.94	137 (45.81)	
Gander	Female	32.12 ± 11.19	719 (46.06)	0.02 (0.43)
	Male	31.74 ± 11.19	363 (46.36)	
Marital status	Marriage	31.54 ± 10.78	421 (44.50)	6.61 (0.10)
	Single	32.31 ± 11.47	666 (47.63)	
Daily duration of use (hour/day)	0–4 h/day	27.18 ± 9.83	274 (27.29)	16.28 (< 0.001)*
	≥ 5 h/day	35.60 ± 10.79	808 (60.29)	
Education level	School student	33.58 ± 12.39	304 (53.23)	5.57 (0.001)*
	Diploma-Undergraduted student	31.87 ± 10.90	266 (45.62)	
	Bachelor’s Degree	31.03 ± 10.90	211 (43.06)	
	Msc degree and above	31.48 ± 10.47	301 (43.	

The comparison between SAS-SV scores in the different categories of age, gender, marital status, and education demonstrated an observed significant difference in the daily duration of smartphone use and the education level variables. Participants with > 5 h/day usage were shown to be addicted to a higher degree than participants with < 4 h/day usage. A *post-hoc* Benfroni test showed a significant difference and higher level of addiction for school students when compared to those with a Bachelor’s degree (*p* = 0.001) and those with a Master’s degree (*p* = 0.005).

For the affected body area, neck pain (43.5%) and eye discomfort (43.3%) were the most prevalent. In contrast, pain at the regions of the shoulder, wrist, finger, and low back were lower at respectively 35.1%, 27.2%, 23.4%, and 10.1%. In order to examine the relationship between smartphone addiction (addicted, non-addicted) and MSDs in different body regions (yes, no), an independent Chi-square test was used. The Chi-square test showed that wrist pain (χ^2^ = 10.2; Phi value = 0.06; *p* = 0.001), finger pain (χ^2^ = 4.8; Phi value = 0.04; *p* = 0.027), shoulder pain (χ^2^ = 37.2; Phi value = 0.12; *p* < 0.001), neck pain (χ^2^ = 75.7; Phi value = 0.18; *p* < 0.001), lumbar pain (χ^2^ = 30.4; Phi value = 0.11; *p* < 0.001), and eye discomfort (χ^2^ = 46.4; Phi value = 0.14; *p* < 0.001), differed significantly between addicted and non-addicted subjects. This indicates that smartphone-addicted participants suffered a higher level of pain in the aforementioned body regions.

Generally, the three most frequently reported functions for using a smartphone were: social networking (89.2%), web surfing (67.36%), and phone calls (65.40%). This usage pattern was the same in both the addicted and non-addicted groups. Further analyses on the pattern of use based on the other demographic characteristics are presented in Table 3.

### Factors associated with smartphone addiction during the pandemic

Smartphone addiction during the pandemic was related to daily duration of smartphone usage (OR = 3.73, 95% CI = 3.11-4.46), social network use (OR = 2.16, 95% CI = 1.58‐2.95), game playing (OR = 1.53, 95% CI = 1.25‐1.87), web surfing (OR = 0.80, 95% CI = 0.65–0.99) and phone calls (OR = 0.75, 95% CI = 0.61‐0.92). Of these, web surfing and phone calls were protective factors against smartphone addiction.

**Table 3:** Patterns of smartphone usage

		Phone call	Web surfing	Email	Game	Social network	News	Camera	Others
Pattern of use in all participants n (%)		1532 (65.40)	1579 (67.36)	512 (21.84)	604 (25.76)	2089 (89.20)	763 (32.55)	1337 (57.03)	514 (21.92)
Gender pattern of use n (%)	Male	523 (67.90)	534 (68.19)	173 (22.10)	193 (24.64)	709 (90.54)	268 (34.22)	438 (55.93)	144 (18.39)
	Female	1009 (64.63)	1045 (66.94)	339 (21.71)	441 (28.25)	1380 (88.40)	495 (31.71)	899 (57.59)	370 (23.70)
Marital status n (%)	Single	874 (62.51)	929 (66.45)	288 (20.60)	377 (26.96)	1242 (92.41)	423 (30.25)	784 (56.08)	324 (23.17)
	Marriage	658 (69.55)	650 (68.71)	224 (23.67)	227 (23.99)	847 (89.53)	340 (35.94)	553 (58.45)	190 (20.10)
Addiction level n (%)	Addicted	665 (61.40)	710 (65.61)	232 (21.44)	332 (30.68)	1007 (93.06)	345 (31.88)	625 (57.76)	252 (23.29)
	Non-addicted	867 (68.70)	869 (68.85)	280 (22.18)	272 (21.55)	1082 (85.73)	418 (33.12)	712 (56.41)	262 (20.76)
Education level n (%)	School student	225 (44.65)	303 (53.06)	43 (7.53)	227 (39.75)	441 (77.23)	92 (16.11)	292 (51.13)	197 (34.50)
	Diploma-Undergraduted student	386 (66.20)	391 (67.06)	96 (16.46)	155 (26.58)	536 (91.93)	203 (34.81)	336 (57.63)	125 (21.44)
	Bachelor’s Degree	365 (74.48)	354 (72.24)	93 (11.77)	112 (22.85)	457 (93.26)	169 (34.48)	297 (60.61)	77 (15.71)
	Msc degrre and above	526 (75.14)	531 (75.85)	280 (40)	110 (15.71)	655 (93.57)	299 (42.71)	412 (58.85)	115 (16.42)

## Discussion

This study aimed to determine the prevalence of smartphone addiction during the COVID-19 pandemic in a general Iranian population, evaluation of the related MSDs, patterns of use, and the risk factors associated with smartphone addiction. Relatively high prevalence of smartphone addiction was found in the general population with the greatest values in school students and the lowest in the higher education to or above a Master’s degree. Social networks were the most common pattern of use with the most involved body regions being the eyes and neck.

The relatively high prevalence of smartphone addiction (46.2%) was observed in different genders, across ages, marital status, and education factors. As smartphones provided the simplest method of internet access during the COVID-19 pandemic, this high prevalence of addiction is most likely a consequence of changes in the routine work schedules across the general population. The global use of ‘lockdowns’ as a public health tool, to reduce social contact and the spread of the virus during the pandemic, forced people into a home-setting and performing of their normal work routine or official tasks through the internet, where a smartphone was the simplest and often only means by which to do so [24]. Additionally, it is recognized that addictive behavior is increased in the presence of trauma [34]. Post-traumatic stress, social isolation, and mandatory work from home during the COVID-19 pandemic, can all be considered as contributors to the high prevalence of smartphone addiction. The previous studies performed during the COVID-19 pandemic showed the general prevalence of internet addiction at 36.7% [22], smartphone addiction in children at 53.3% [24], university students at 37.4% [23], and medical students at 52.8% [25]. These numbers are substantially higher than that reported in a recent meta-analysis of smartphone addiction pre-COVID-19 at 23% [19] as highlighted earlier. In contrast, our results showed addiction levels at 46.2%, which is within the range of these reported pandemic findings and twice that of the reported pre-pandemic levels.

There was no statistically significant difference between smartphone addiction prevalence when categorized by gender, age, and marital status. There was a significant difference in the daily duration of smartphone use and the education level variables. Similar findings for smartphone addiction were also reported in a previous study [35] as were the levels of addiction during Italian lockdowns at 46.7% [26]. It appears that with lockdowns, the imposed lifestyle, and consequential social restrictions along with limited face-to-face interactions, caused a tendency for increased smartphone use across all age and gender classifications [20, 36].

There was reported an increase in general usage in both addicted and non-addicted groups relative to pre-pandemic levels [20, 37], similar to that reported in China, Germany, and Italy with respective increases of 46.8% [38], 53.2% [39] and 56% [26] for internet and online activities during the COVID-19 lockdown. This significant increase in the level of reported smartphone usage during COVID-19 is consistent when compared to the reported pre-pandemic period [37], though the reported percentage of confirmed increase has varied [40, 41]. The leading reason for such addiction appears to be that the smartphone remains a critical and common source of communication, information, and entertainment [42]. During a period of restriction and isolation, such as lockdowns during the COVID-19 pandemic, there is a clear need to make connections with family and friends, to participate in learning programs, access entertainment, and seek social support, which all contribute to the subsequent increase in smartphone usage.

Smartphone addiction has been associated with musculoskeletal pain in different body regions for some time [43]. The neck, shoulder, upper back, elbow, wrist/hand, low back, hip/thigh, knee, and ankle/foot are reported as the most common regions affected. This study used sections of the ENMQ to investigate body region pain most frequently reported during smartphone usage and an additional dichotomous question on perceived eye strain. Similar results have been reported that implicate the neck [11, 14, 33, 44], eyes [11, 33, 45], and upper limb components of the wrist, fingers, and shoulders [11, 14, 44, 45] as the most involved [46]. Similar to our findings, a systematic review of MSD and pathologies related to mobile phone usage noted the body regions most often affected by MSDs were the neck (55.8–89.9%), followed by the shoulders (37.8–71.6%), hands/wrists (13–32%), and elbows (14.1–15%) in a general population [43].

Individuals will use smartphones in poor postures such that neck flexion is sustained for extended periods when the elbows are not supported. It is recognized that flexed postures of the neck and back are increased significantly during smartphone usage in sitting [47]. Similarly, associated repetitive fingers and wrist movements will increase load and worsen these conditions, while trapezius muscle activity increases and imposes further neck and shoulder muscle load [43, 48]. Other exacerbating factors for upper limb and neck MSDs include holding the phone with one hand [49], a static posture, and no external smartphone support [14, 50]. When smartphones are constantly used in poor postures, without support, over long periods of time, musculoskeletal pain will gradually present. Moreover, concurrent with pain, eye discomfort was also reported by our study’s participants. The high prevalence of dry-eye syndrome in smartphone users has been recognized in previous research as up to 42.1% [14].

Within our study, smartphone usage was most commonly for social networking, web surfing, and phone calls. Similar findings were reported where 80.7% of total usage was accounted for by internet-chating (42.5%) and web surfing (38.2%) [14]. Due to the social isolation imposed by the COVID-19 pandemic lockdowns, participants used their smartphones to enable a virtual-connect with friends and family to discuss daily activities. Further, social networking was used as a coping technique to resolve the feelings of loneliness. This logically explains the levels of reported smartphone activity through social networking sites [37, 51]. Web surfing and internet searches are the second most often reported activity with a possible explanation being the natural tendency to seek pandemic-related information, including prevention of disease or infection [37]. Prior to COVID-19, phone calls were the primary smartphone usage reported, but our results showed this decreased during the pandemic. This decrease is likely to have been due to an increase in the use of other apps for connection, namely social networking, web-conferencing, and video-calls [37]. The necessity to remain at home with family would consequently further reduce the phone calls to these close family members [39]. Our study showed that for an Iranian population smartphone gaming was not a prevalent activity during the pandemic, which is in contrast to previous research [39, 52]. A confounding factor may have been that in the current study, only smartphone gaming was recorded, while other gaming devices such as laptops, PCs, or consoles were not.

Previous research has evaluated the different factors related to smartphone addiction which included sociodemographic characteristics, pattern of use, and both physical and mental health status. Our findings showed that the significant related factors for smartphone addiction were respectively usage related to the daily duration, social networking, web surfing, phone calls, and game-play. Several studies have reported that longer usage time corresponded to higher addiction [53–56] and that the cut-off is in the order of > 4 h/day [14, 57], which approximated our findings. Similarly, social networking and game-play were reported as related factors for smartphone addiction [58].

Considerations of age, gender, marital status, and education in this study were not found to be significant contributors to smartphone addiction during the COVID-19 pandemic. The findings on these parameters as definitive contributors to smartphone addiction is inconsistent. Some studies determined there was a gender difference [59] while others did not [60]. To explore these reported gender variations the purposes of use may provide an explanation. Previous studies have reported that males spend more time on games, cybersex, and gambling than females; while females engage more in chatting, messenger systems, and blogging [61]. In contrast, this study found no gender variation for the smartphone’s purposes of use during the pandemic. Potentially these may be culturally biased and further research in this area is required.

The consideration of education level is an important finding from this study. Although the education level was not significant in smartphone addiction, it was noted that school students experienced the highest levels of addictive behavior. The requirement for many students to have virtual education resulted in the need for a smartphone as the simplest and most economical immediate solution to both lockdown and remote learning. When the degree of free time available during the pandemic is taken into account, despite the time required for education, students occupied their spare time with smartphones for personal reasons such as communication, fun, and entertainment.

### Strengths and weakness

The strengths of this cross-sectional study were that this was, to our knowledge, the first reported Iranian population study investigating the prevalence of smartphone addiction during the COVID-19 pandemic. The findings may also assist with comparisons to other countries and global communities to facilitate understanding of the overall global effect. The participants were from a general population across the age range and showed diversity in gender along with marital and educational status. Further, the population size and overall demographic analysis may increase the generalizability of the findings. Due to the cross-sectional nature of the study, there is a limitation on the generalizability of the results to periods other than during significant social restrictions, as occurred during the COVID-19 pandemic.

Other limitations include that the data is self-reported, and consequently subject to recall and reporting bias, as participants may have omitted information and either deliberately or unconsciously reduced or embellished their smartphone interactions. Further, other internet access devices such as PCs, consoles, and Tablets, were not accounted for which may affect the findings for some participation levels, particularly that of game playing. The potential bias due to the higher female participation must also be acknowledged. A further consideration that may affect generalizability is that of occupation. This may include whether or not the individual had lockdown-exemption, such as a critical reason or occupation that reduced the lockdown compliance such as being first responders or emergency workers.

## Conclusions

In this general population during the period of the COVID-19 pandemic and public health lockdowns the level of smartphone addiction was found to be more prevalent than that reported in pre-pandemic studies. The most common usage purposes were social networking, web surfing, and phone calls. The significant increase in usage duration indicated an overuse that led to MSDs in the neck, wrist, and shoulders, along with concurrent eye discomfort. The parameters of duration of daily use, social networking, and gaming were significant predictors of smartphone addiction. Such addiction has recognized associations with negative impacts on physical and mental health. Consequently, it is essential to plan for early intervention and the provision of strategies for the general population to mitigate the risks of smartphone use, particularly during periods of required social restriction.

## Data Availability

The data used in this article can be made available by the corresponding author upon request.

## Abbreviations

COVID-19: Coronavirus disease 2019
SAS-SV: Smartphone Addiction Scale short Version
ENMQ: Extended Nordic Musculoskeletal Questionnaire
WHO: World Health Organization
MSDs: Musculoskeletal Disorders
LR: logistic regression
SD: Standard Deviation
OR: Odds Ratio
CI: Confidence Interval
